# Supplementary material for: Two Pairs of 7,7′-Cyclolignan Enantiomers with Anti-Inflammatory Activities from Perilla frutescens
Source: Molecules. 2022 Sep 18;27(18):6102. doi: 10.3390/molecules27186102 (PMC9506341; doi:10.3390/molecules27186102)
Supplement: Supplementary file 1 [file molecules-27-06102-s001.zip › molecules-1914835-supplementary.pdf]

Supplementary Materials

Two pairs of 7,7′-cyclolignan enantiomers with anti-inflammatory activities from *Perilla frutescens*

Jing Zuo [1,2,3,†], Tian-Hao Zhang [1,2,3,†], Liang Xiong [1,2,3], Lu Huang [1,2,3], Cheng Peng [1,2], Qin-Mei Zhou[1,2,3,4,*], and Ou Dai[1,2,3,*]

[1]*State Key Laboratory of Southwestern Chinese Medicine Resources, School of Pharmacy, Chengdu University of Traditional Chinese Medicine, Chengdu, 611137, China*

[2]*School of Pharmacy, Chengdu University of Traditional Chinese Medicine, Chengdu 611137, China*

[3]*Institute of Innovative Medicine Ingredients of Southwest Specialty Medicinal Materials, School of Pharmacy, Chengdu University of Traditional Chinese Medicine, Chengdu 611137, China*

[4]*Innovative Institute of Chinese Medicine and Pharmacy, Chengdu University of Traditional Chinese Medicine, Chengdu 611137, China*

[*] Corresponding authors. *E-mail address*: zhqmyx@sina.cn (Q.-M.Z.); daiou@cdutcm.edu.cn (O.D.)

[†] Both authors contributed equally to this work.

The List of Contents

No.	Content	Page
1	**General experimental details**	S1
2	**ECD calculation of compound 1**	S1
3	**Figure S1.** *ω*B97XD/DGDZVP optimized 15 conformers of (7*S*,8*S*,7′*R*,8′*S*)-**1**	S2
4	**Table S1.** Energy analysis for the conformers of (7*S*,8*S*,7′*R*,8′*S*)-**1**	S2
5	**ECD calculation of compound 2**	S3
6	**Table S2.** Energy analysis for the conformers of (7*R*,8*S*,7'*R*,8'*S*)-**2**	S3
7	**Figure S2.** *ω*B97XD/DGDZVP optimized 5 conformers of (7*R*,8*S*,7'*R*,8'*S*)-**2**	S4
8	**Figure S3.** The (+)-HR-ESI-MS spectroscopic data of compound **1**	S5
9	**Figure S4.** The IR spectrum of compound **1** in CH_3CN	S6
10	**Figure S5.** The ^1H NMR spectrum of compound **1** in $CDCl_3$	S7
11	**Figure S6.** The ^{13}C NMR spectrum of compound **1** in $CDCl_3$	S8
12	**Figure S7.** The HSQC spectrum of compound **1** in $CDCl_3$	S9
13	**Figure S8.** The ^1H-^1H COSY spectrum of compound **1** in $CDCl_3$	S10
14	**Figure S9.** The HMBC spectrum of compound **1** in $CDCl_3$	S11
15	**Figure S10.** The NOESY spectrum of compound **1** in $CDCl_3$	S12
16	**Figure S11.** The (+)-HR-ESI-MS spectroscopic data of compound **2**	S13
17	**Figure S12.** The IR spectrum of compound **2** in CH_3CN	S14
18	**Figure S13.** The ^1H NMR spectrum of compound **2** in $CDCl_3$	S15
19	**Figure S14.** The ^{13}C NMR spectrum of compound **2** in $CDCl_3$	S16
20	**Figure S15.** The HSQC spectrum of compound **2** in $CDCl_3$	S17
21	**Figure S16.** The ^1H-^1H COSY spectrum of compound **2** in $CDCl_3$	S18
22	**Figure S17.** The HMBC spectrum of compound **2** in $CDCl_3$	S19
23	**Figure S18.** The NOESY spectrum of compound **2** in $CDCl_3$	S20
24	**Table S3.** The original western blots in three repetitions for Figure 7 in the paper	S21

General experimental details

Optical rotations were measured using a Rudolph Autopol-I automatic polarimeter (Rudolph Research Analytical, USA). An Agilent Cary 600 FT-IR microscope was used to measure the IR spectra (Agilent Technologies Inc., CA, USA). ECD spectra were recorded using an Applied photophysics Chirascan and Chirascan-plus circular dichroism spectrometer (Applied Photophysics Ltd., Leatherhead, England). X-ray diffraction analyses were performed on a Bruker D8 Quest diffractometer with an APEX-II CCD (Bruker Corporation, MA, USA). HR-ESI-MS data were obtained using an Agilent P/N G1969-90010 instrument (Agilent Technologies Inc., CA, USA). NMR data were recorded by a Bruker Avance-III NEO-600 NMR spectrometer (Bruker Corporation, Billerica, MA, USA) and solvent peaks were used as internal standards. TLC was performed using glass-precoated silica gel GF254 plates (Qingdao Marine Chemical Inc., Qingdao, China). Column chromatography separations were performed on Silica gel (200–300 mesh, Yantai Institute of Chemical Technology, Yantai, China) and Sephadex LH-20 columns (Amersham Pharmacia Biotech AB, Uppsala, Sweden). HPLC separations were carried out using an Agilent 1100 instrument. A Zorbax SB-C18 column (250 × 9.4 mm^2, 5 μm) was employed for semipreparative reversed-phase (RP) HPLC (Agilent Technologies Inc., CA, USA), and a Chiralpak IG column (250 × 4.6 mm^2, 5 μm) was used for enantiomeric separation (Daicel Chiral Technologies Co., Ltd., Shanghai, China).

ECD Calculations of (7*S*,8*S*,7′*R*,8′S)-1 and (7*R*,8*R*,7′*S*,8′*R*)-1

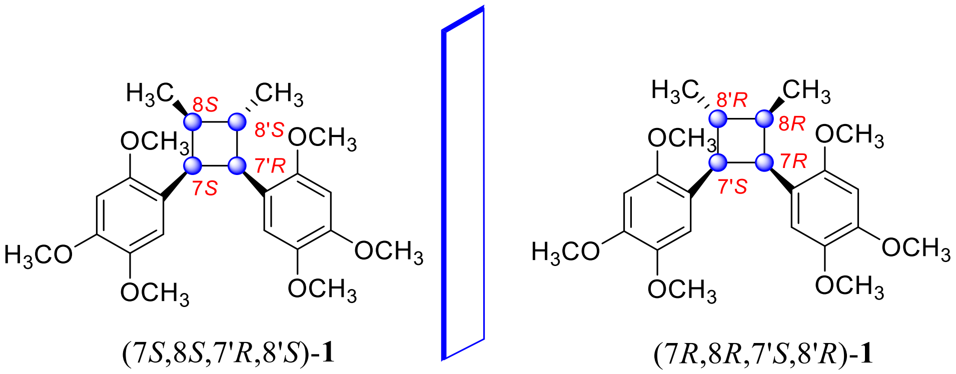

(7*S*,8*S*,7'*R*,8'*S*)-**1** (7*R*,8*R*,7'*S*,8'*R*)-**1**

Conformation searches based on molecular mechanics with MMFF94s force field were performed for (7*S*,8*S*,7′*R*,8′*S*)-**1** and gave 15 conformers with populations higher than 1% [37,38]. The selected conformers were optimized using DFT at B3LYP/6-31G (d) level in vacuum with the Gaussian 16 program (Table S1) [39]. Then,

the B3LYP/6-31G (d)-optimized conformers were reoptimized at the *ω*B97XD/DGDZVP level in acetonitrile (Table S1). ECD computations for the *ω*B97XD/DGDZVP-optimized conformers (Figure S1) were carried out at the CAM-B3LYP/DGDZVP level in acetonitrile [40]. According to the Boltzmann distribution theory and their relative Gibbs free energy (ΔG), the ECD spectrum for (7*S*,8*S*,7′*R*,8′*S*)-**1** was generated using SpecDis 1.71 with σ = 0.25 eV and a UV shift of +19 nm [41]. The corresponding theoretical ECD spectrum of (7*R*,8*R*,7′*S*,8′*R*)-**1** was depicted by inverting that of (7*S*,8*S*,7′*R*,8′*S*)-**1**.

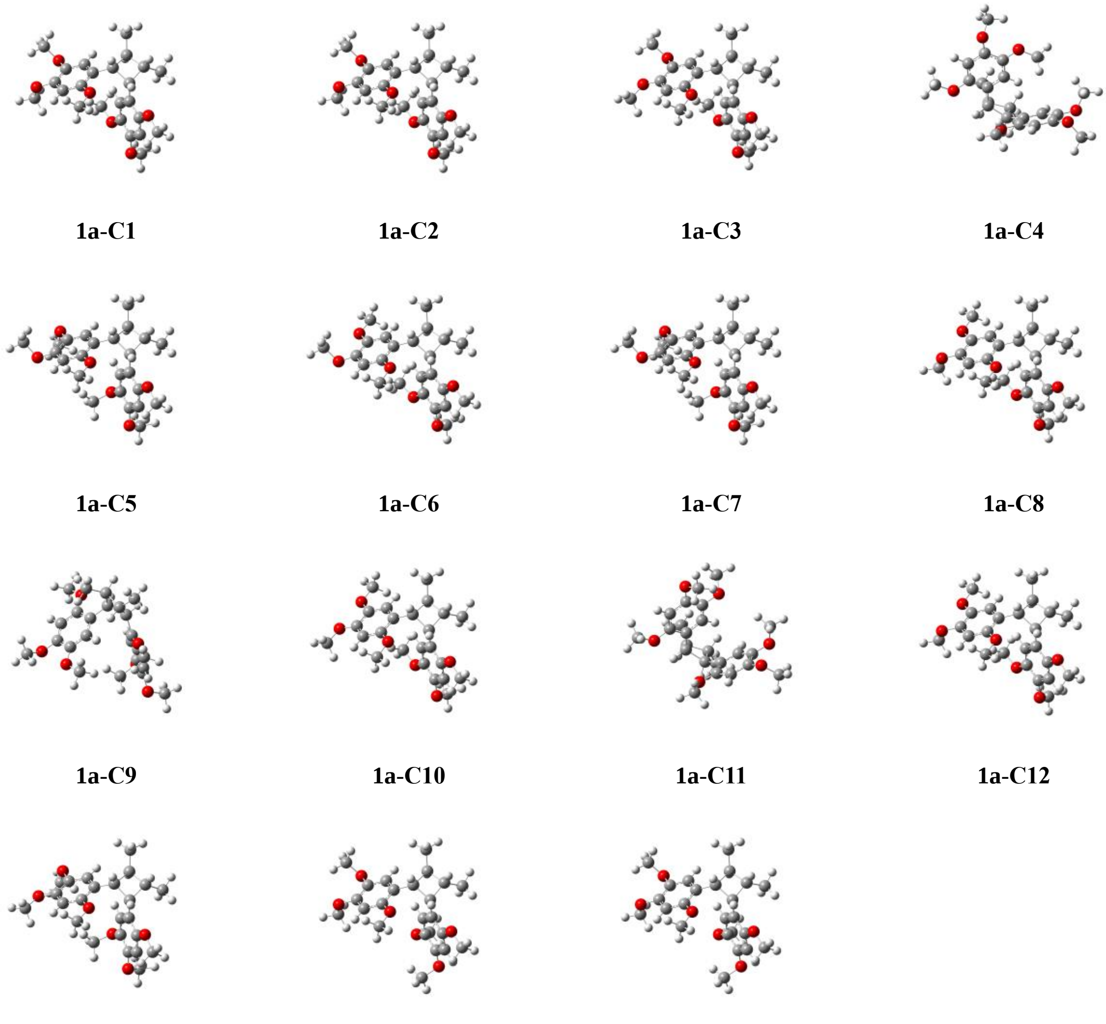

Figure S1. *ω*B97XD/DGDZVP optimized 15 conformers of (7*S*,8*S*,7′*R*,8′*S*)-**1** in CH_3CN.

Table S1. Energy analysis for the conformers of (7*S*,8*S*,7′*R*,8′*S*)-**1** in CH_3CN.

Conf.	MMFF energy	B3LYP/6-31G(d) Gibbs free energy (298.15 K)			*ω*B97XD/DGDZVP Gibbs free energy (298.15 K)		
	ΔE (Kcal/mol)	G (Hartree)	ΔG (Kcal/mol)	Boltzmann Distribution	G (Hartree)	ΔG (Kcal/mol)	Boltzmann Distribution
**1a-C1**	0.0000	-1384.600068	0.0000	0.118	-1384.323558	0.0000	0.093
**1a-C2**	0.0018	-1384.600071	-0.0020	0.118	-1384.323564	-0.0040	0.093
**1a-C3**	0.0245	-1384.600411	-0.2150	0.169	-1384.323881	-0.2030	0.13
**1a-C4**	0.1184	-1384.598715	0.8490	0.028	-1384.321887	1.0490	0.016
**1a-C5**	0.1249	-1384.599695	0.2340	0.079	-1384.324193	-0.3980	0.182

**1a-C6**	0.1511	-1384.599026	0.6540	0.039	-1384.322923	0.3980	0.047
**1a-C7**	0.2670	-1384.599696	0.2330	0.079	-1384.324189	-0.3960	0.181
**1a-C8**	0.4723	-1384.599185	0.5540	0.046	-1384.323336	0.1390	0.073
**1a-C9**	0.5821	-1384.596061	2.5140	0.002	-1384.320162	2.1310	0.003
**1a-C10**	0.9350	-1384.599629	0.2750	0.074	-1384.322575	0.6170	0.033
**1a-C11**	0.9370	-1384.598573	0.9380	0.024	-1384.321703	1.1640	0.013
**1a-C12**	0.9588	-1384.599184	0.5550	0.046	-1384.323281	0.1740	0.069
**1a-C13**	1.1240	-1384.599947	0.0760	0.103	-1384.323136	0.2650	0.059
**1a-C14**	1.1570	-1384.598976	0.6850	0.037	-1384.320667	1.8140	0.004
**1a-C15**	1.1778	-1384.598977	0.6850	0.037	-1384.320645	1.8280	0.004

ECD Calculations of (7*R*,8*S*,7′*R*,8′*S*)-2 and (7*S*,8*R*,7′*S*,8′*R*)-2

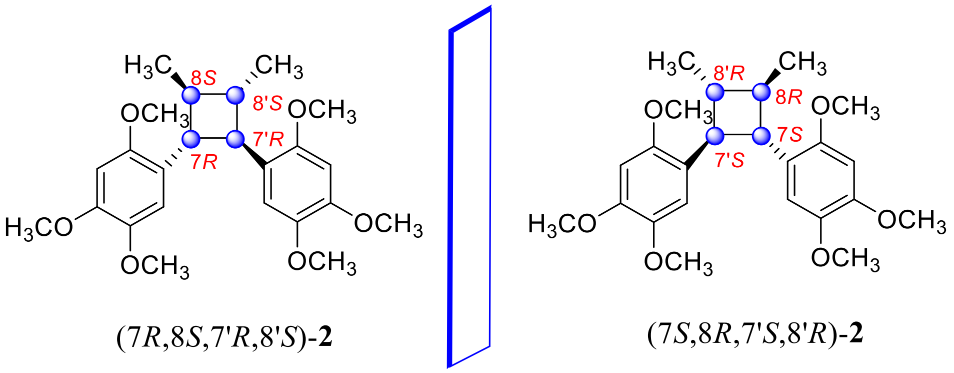

(7*R*,8*S*,7'*R*,8'*S*)-**2** (7*S*,8*R*,7'*S*,8'*R*)-**2**

Conformation searches based on molecular mechanics with MMFF94s force field were performed for (7*R*,8*S*,7′*R*,8′*S*)-**2** and gave 5 conformers with populations higher than 1% [37,38]. The selected conformers were optimized using DFT at B3LYP/6-31G (d) level in vacuum with the Gaussian 16 program (Table S2) [39]. Then, the B3LYP/6-31G (d)-optimized conformers were reoptimized at the *ω*B97XD/DGDZVP level in acetonitrile (Table S2). ECD computations for the *ω*B97XD/DGDZVP-optimized conformers (Figure S2) were carried out at the CAM-B3LYP/DGDZVP level in acetonitrile [40]. According to the Boltzmann distribution theory and their relative Gibbs free energy (ΔG), the ECD spectrum for (7*R*,8*S*,7′*R*,8′*S*)-**2** was generated using SpecDis 1.71 with σ = 0.25 eV and a UV shift of +4 nm [41]. The corresponding theoretical ECD spectrum of (7*S*,8*R*,7′*S*,8′*R*)-**2** was depicted by inverting that of (7*R*,8*S*,7′*R*,8′*S*)-**2**.

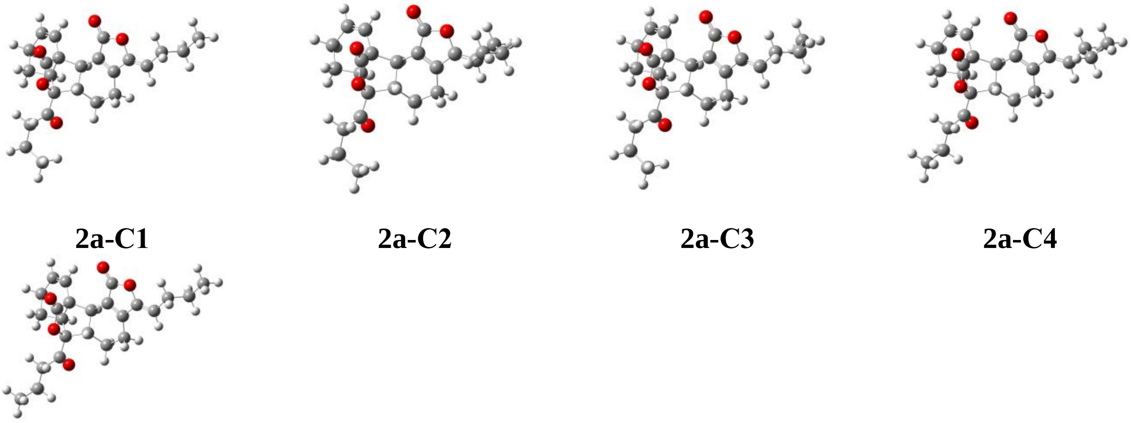

2a-C1 **2a-C2** **2a-C3** **2a-C4**

2a-C5

Figure S2. *ω*B97XD/DGDZVP optimized 5 conformers of (7*R*,8*S*,7′*R*,8′*S*)-**2** in CH_3CN.

Table S2. Energy analysis for the conformers of (7*R*,8*S*,7′*R*,8′*S*)-**2** in CH_3CN.

**Conf.**	**MMFF energy**	**B3LYP/6-31G(d) Gibbs free energy (298.15 K)**			***ω*B97XD/DGDZVP Gibbs free energy (298.15 K)**		
	**ΔE (Kcal/mol)**	**G (Hartree)**	**ΔG (Kcal/mol)**	**Boltzmann Distribution**	**G (Hartree)**	**ΔG (Kcal/mol)**	**Boltzmann Distribution**
**2a-C1**	0.00000	-1384.607238	0.0000	0.18	-1384.324698	0.158628197	0.0000
**2a-C2**	0.12260	-1384.607207	0.0190	0.175	-1384.323651	0.052305594	0.6570
**2a-C3**	0.17120	-1384.60725	-0.0080	0.183	-1384.324554	0.136179331	0.0900
**2a-C4**	0.22080	-1384.607372	-0.0840	0.208	-1384.325368	0.322638516	-0.4200
**2a-C5**	0.23810	-1384.607562	-0.2030	0.254	-1384.32539	0.330248362	-0.4340

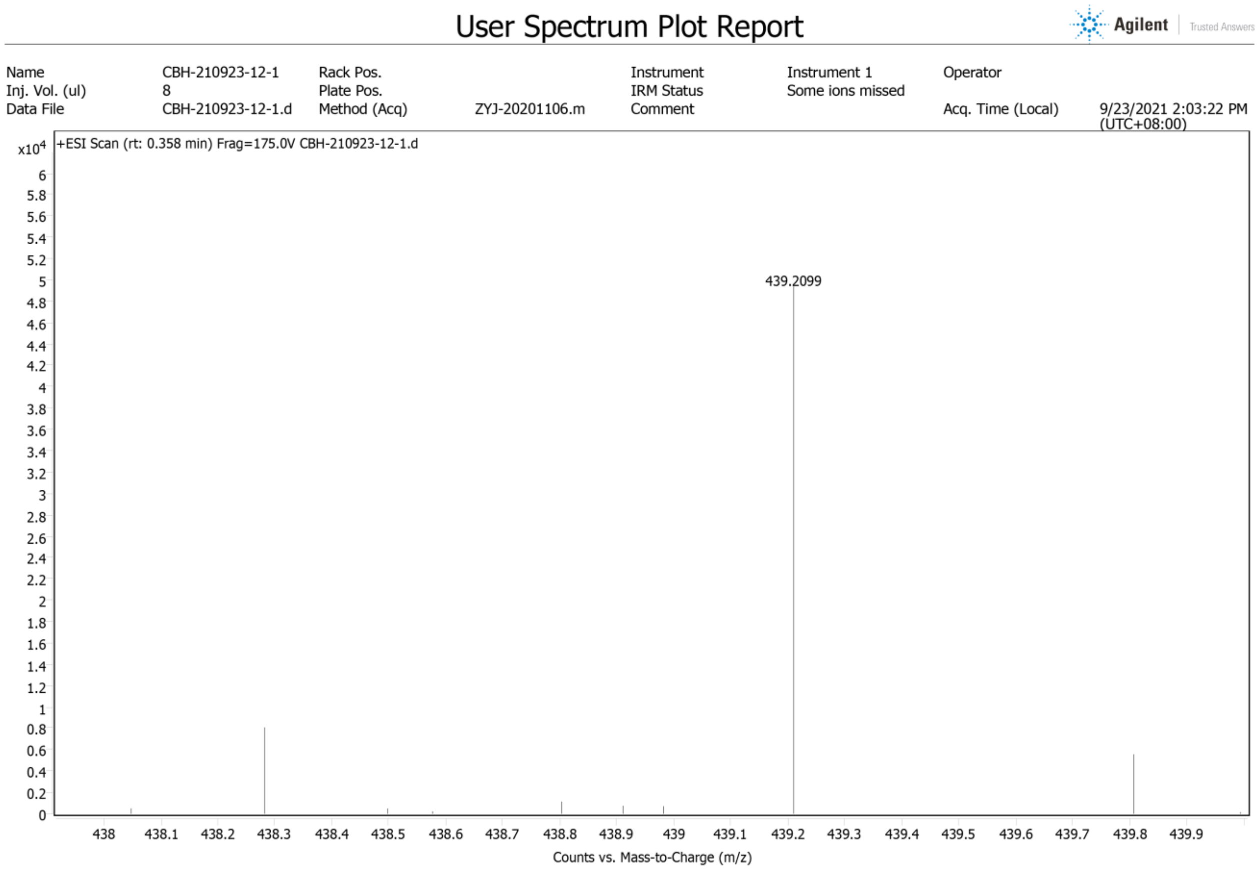

Figure S3. The (+)-HRESIMS spectroscopic data of compound **1**

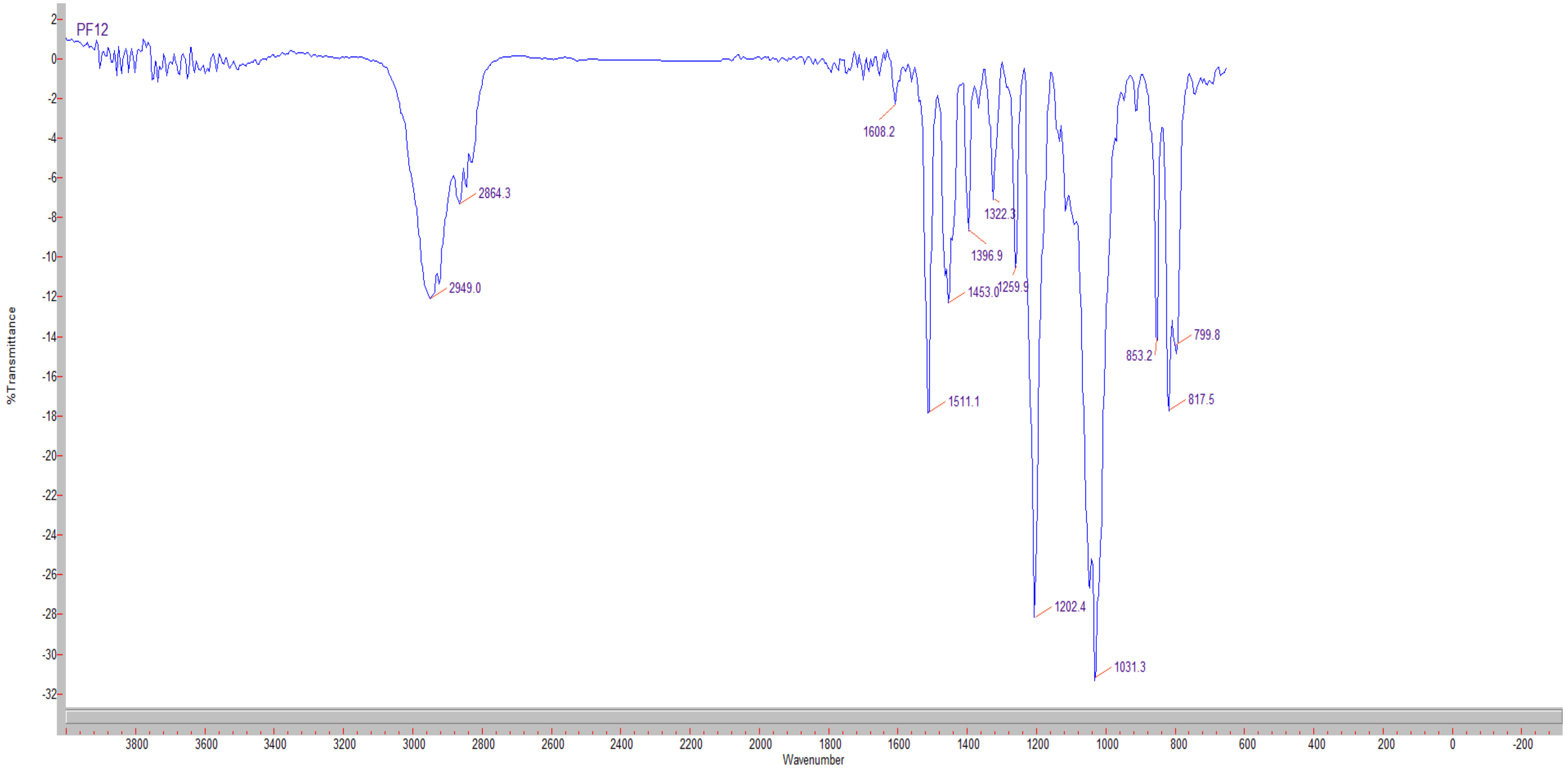

Figure S4. The IR spectrum of compound **1**

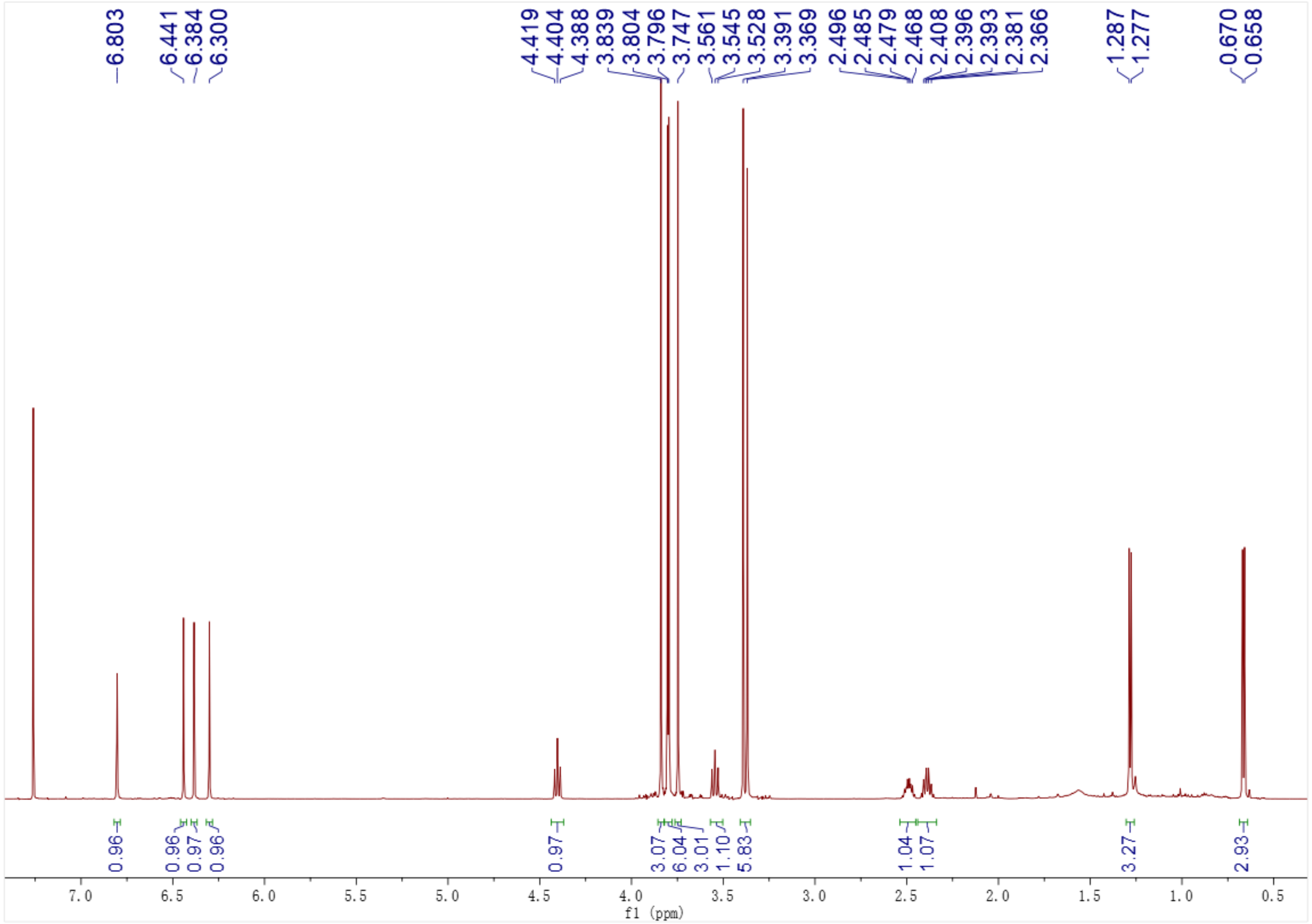

Figure S5. The ^{1}H NMR spectrum of compound **1** in $CDCl_3$ (600 MHz)

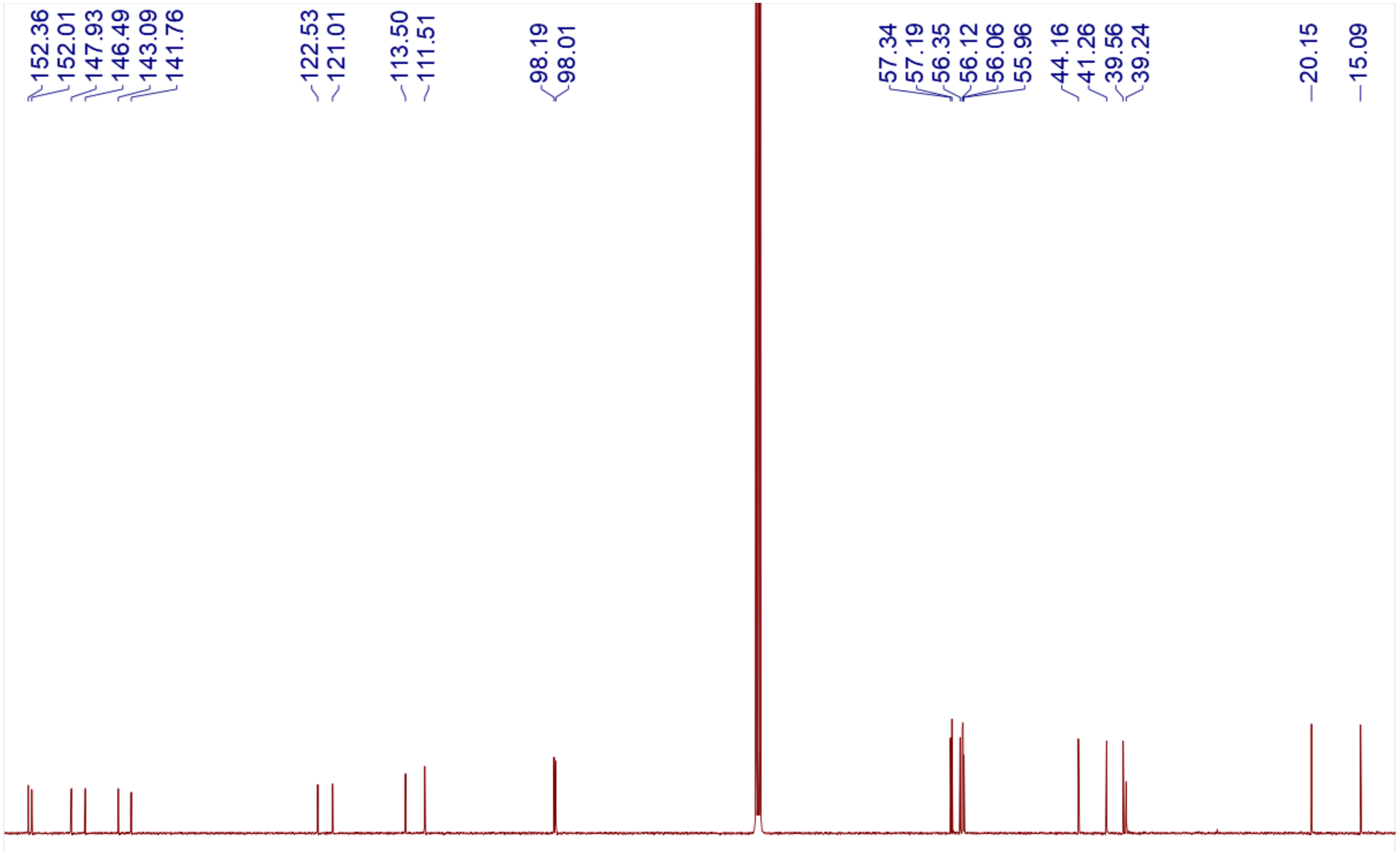

Figure S6. The ^{13}C NMR spectrum of compound **1** in $CDCl_3$ (150 MHz)

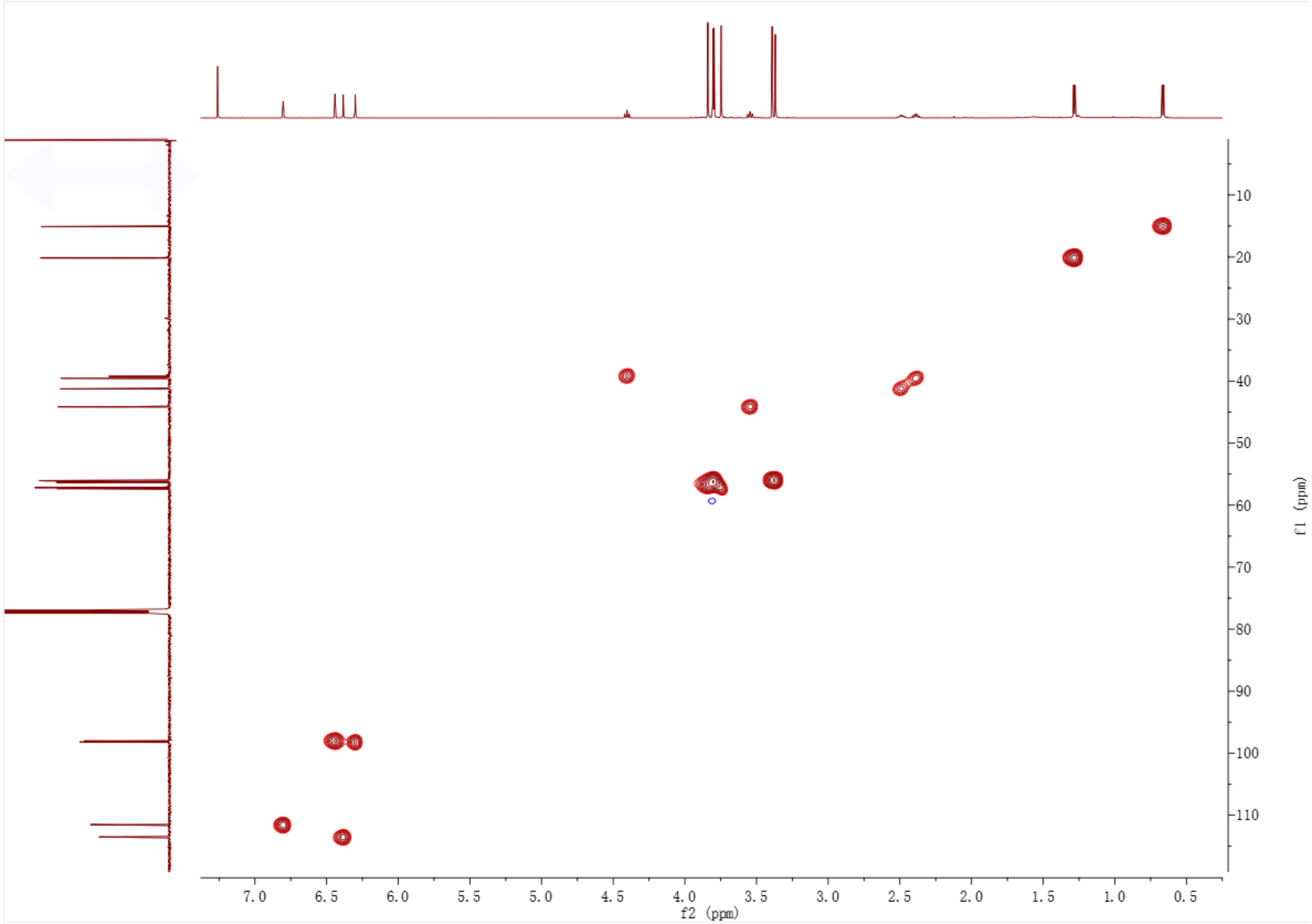

Figure S7. The HSQC spectrum of compound **1** in $CDCl_3$

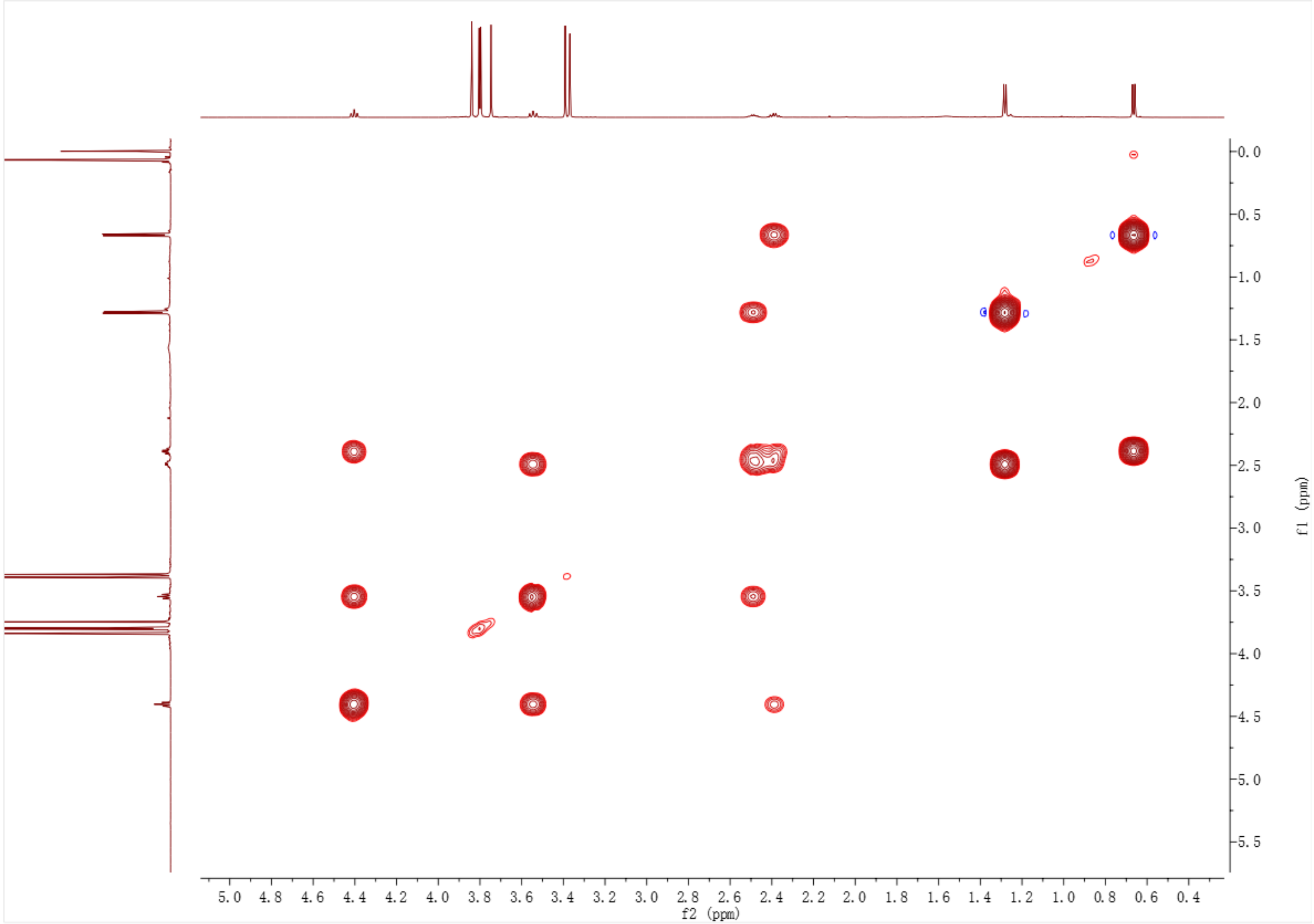

Figure S8. The ^{1}H-^{1}H COSY spectrum of compound **1** in $CDCl_3$

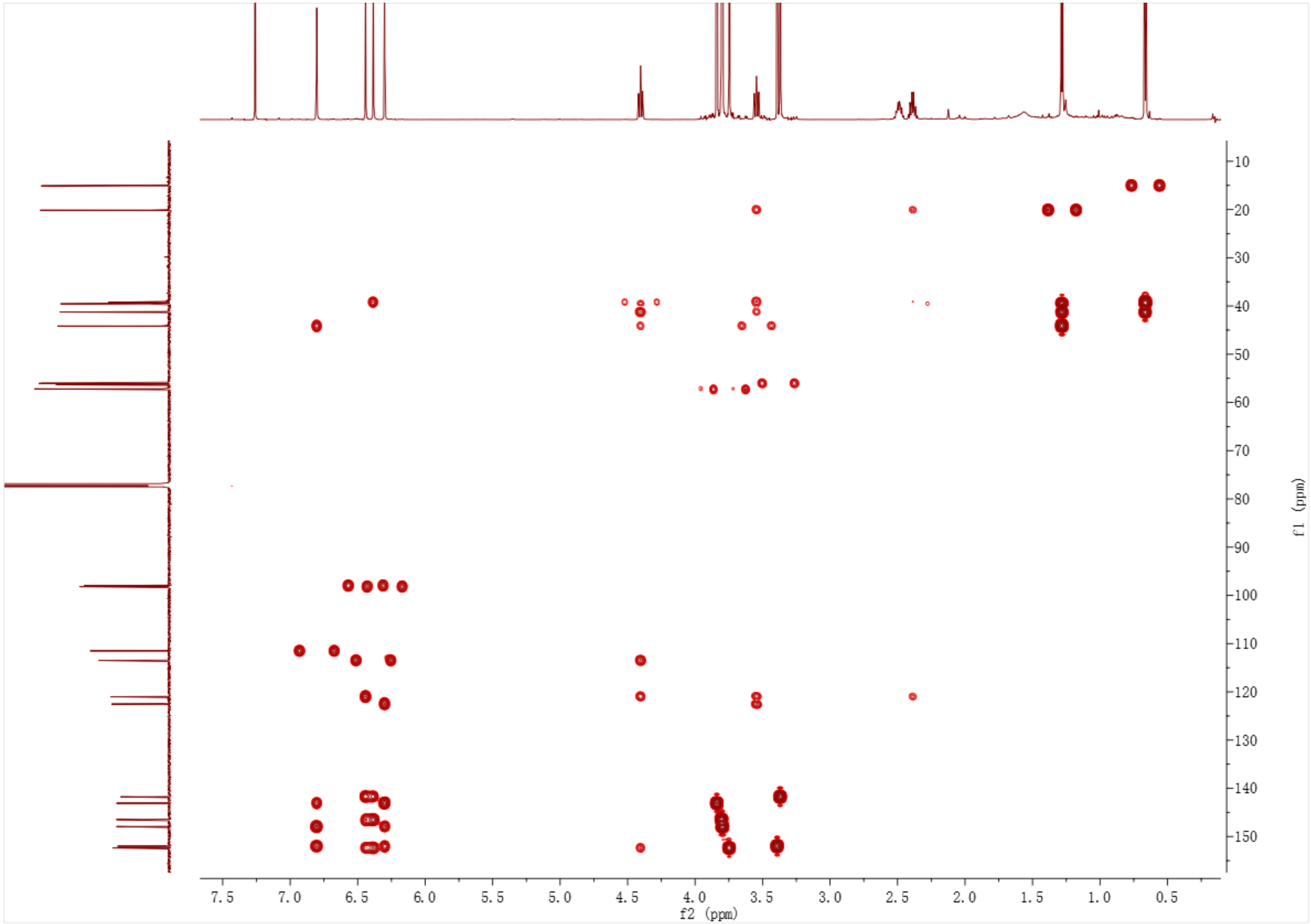

Figure S9. The HMBC spectrum of compound **1** in $CDCl_3$

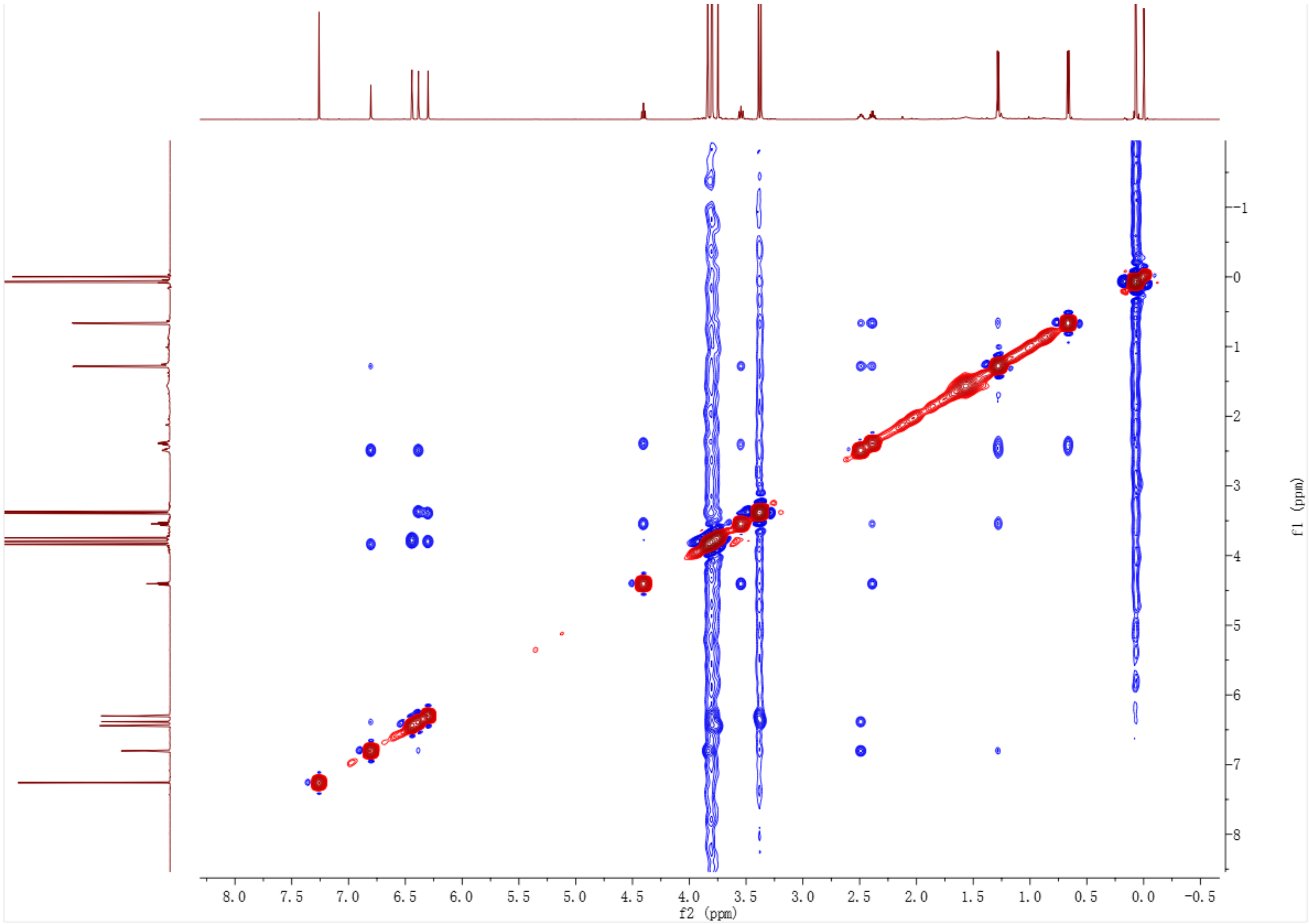

Figure S10. The NOESY spectrum of compound **1** in $CDCl_3$

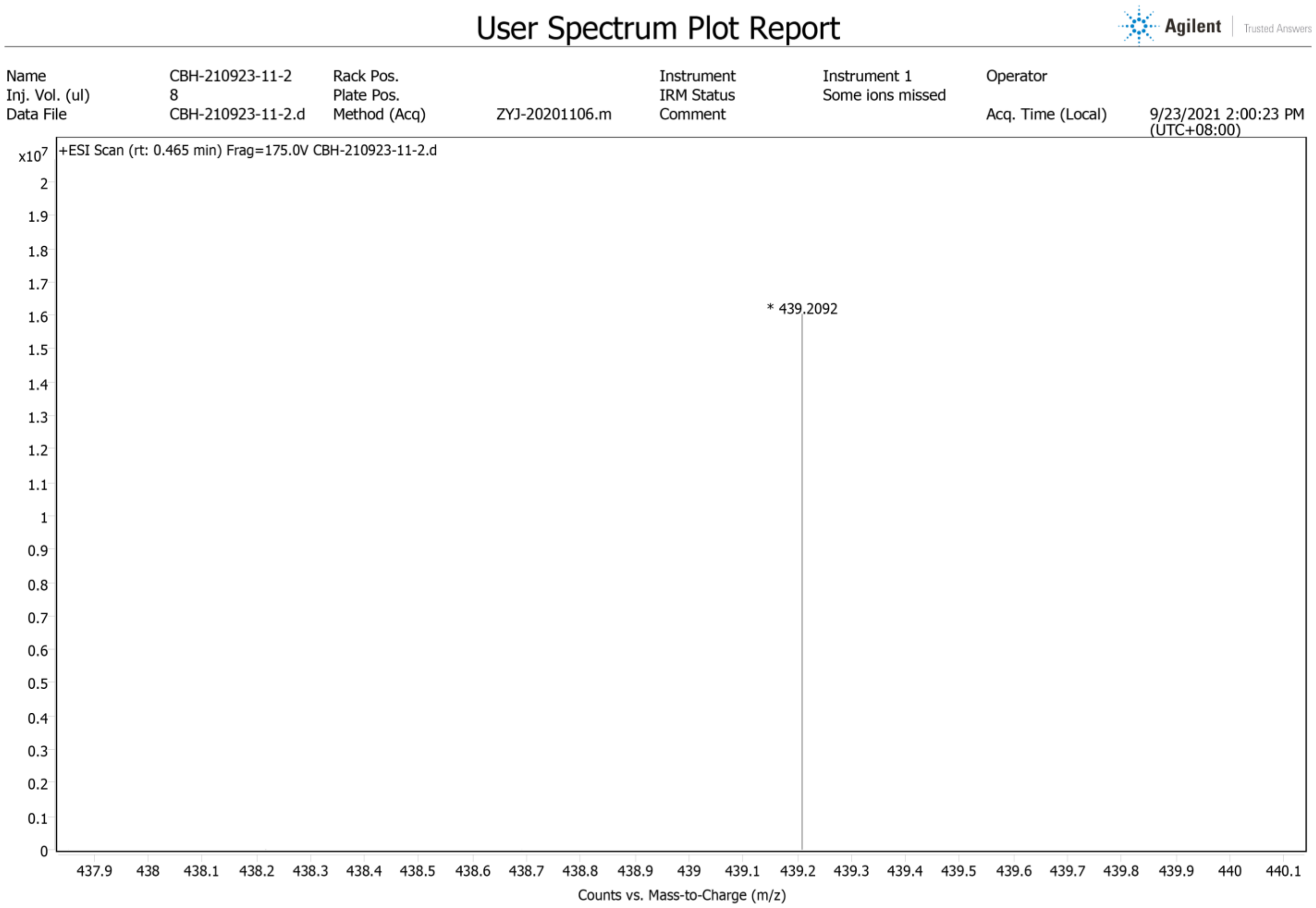

Figure S11. The (+)-HRESIMS spectroscopic data of compound **2**

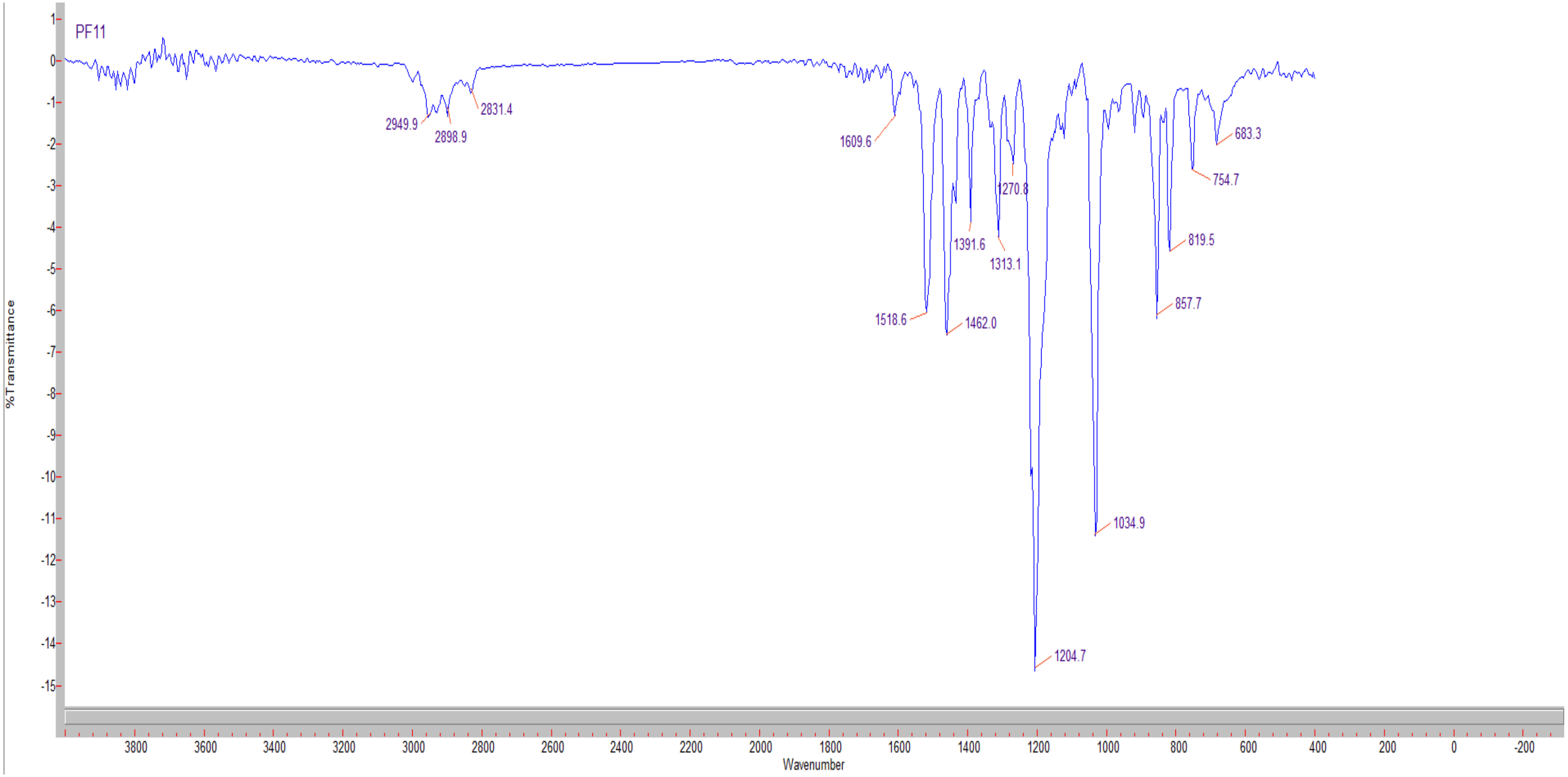

Figure S12. The IR spectrum of compound **2**

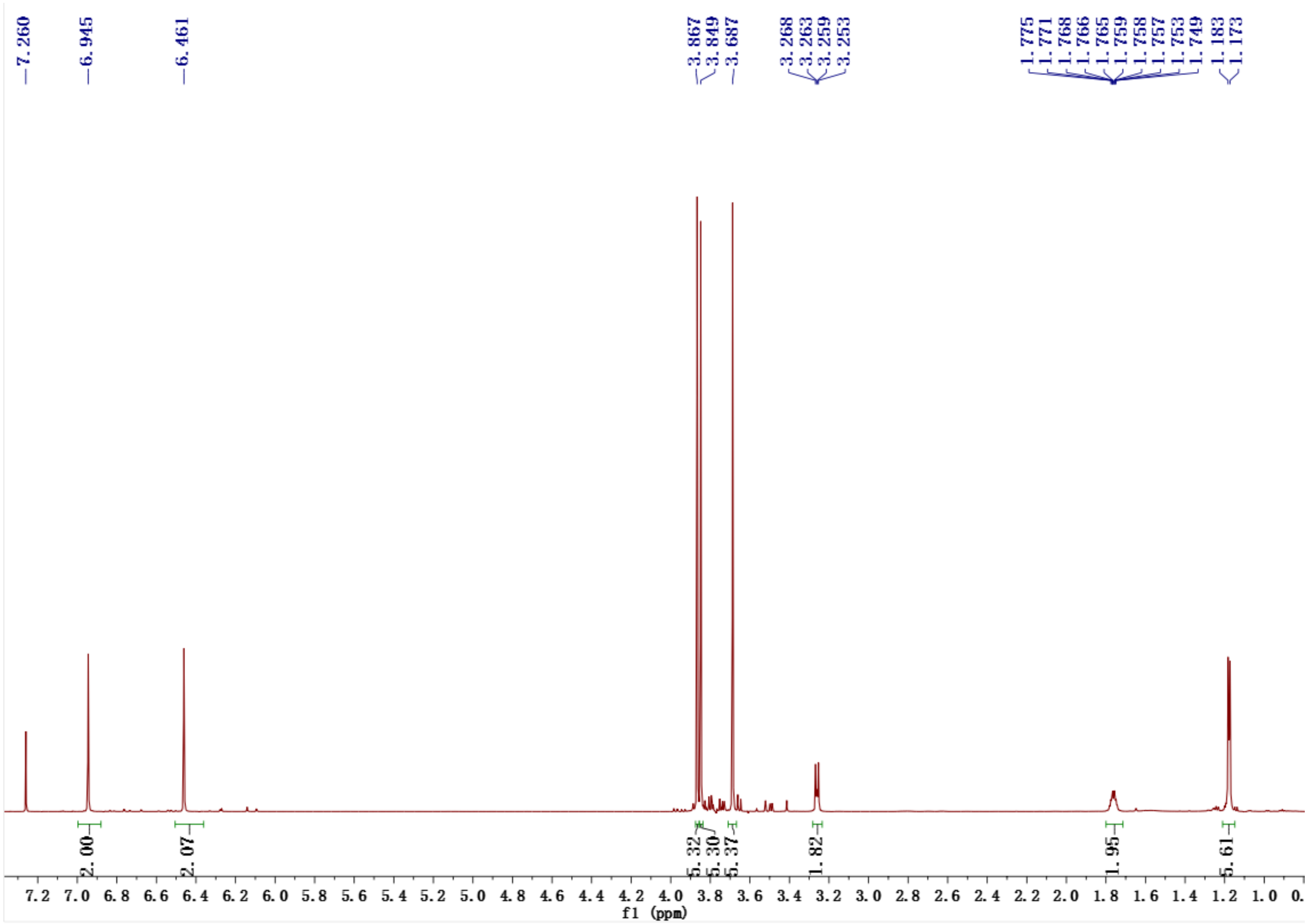

Figure S13. The ^{1}H NMR spectrum of compound **2** in $CDCl_3$ (600 MHz)

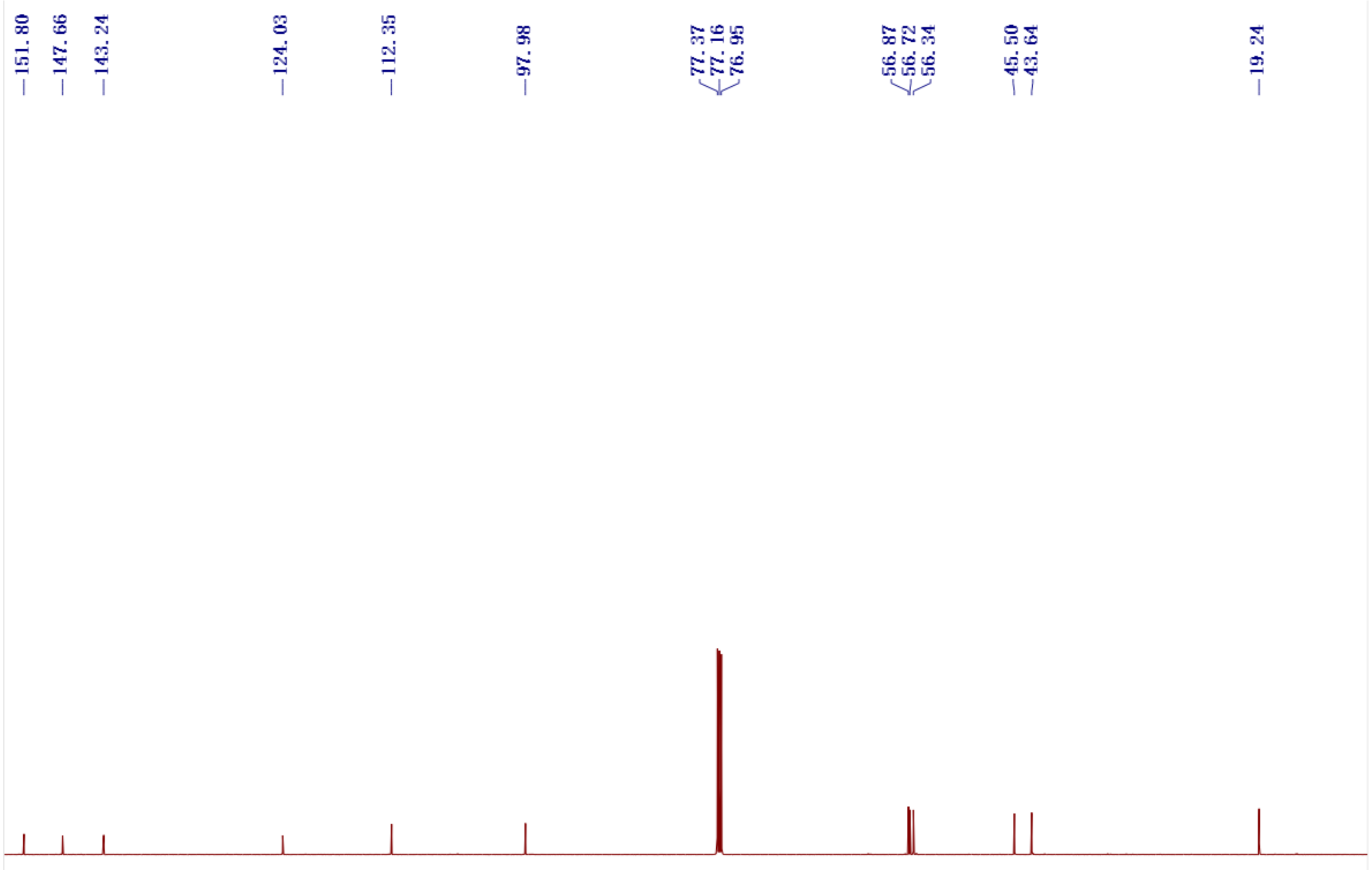

Figure S14. The ^{13}C NMR spectrum of compound **2** in $CDCl_3$ (150 MHz)

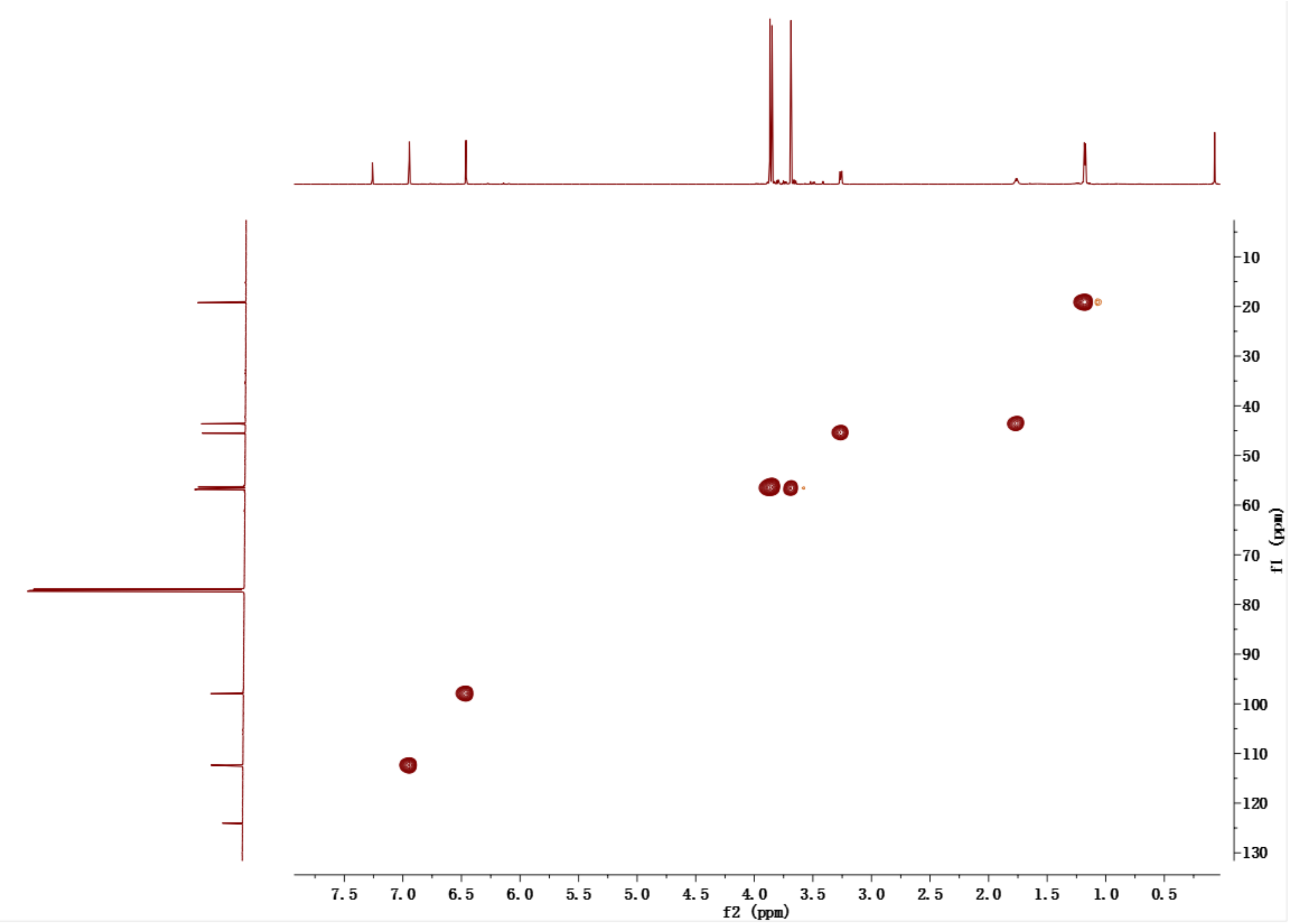

Figure S15. The HSQC spectrum of compound **2** in $CDCl_3$

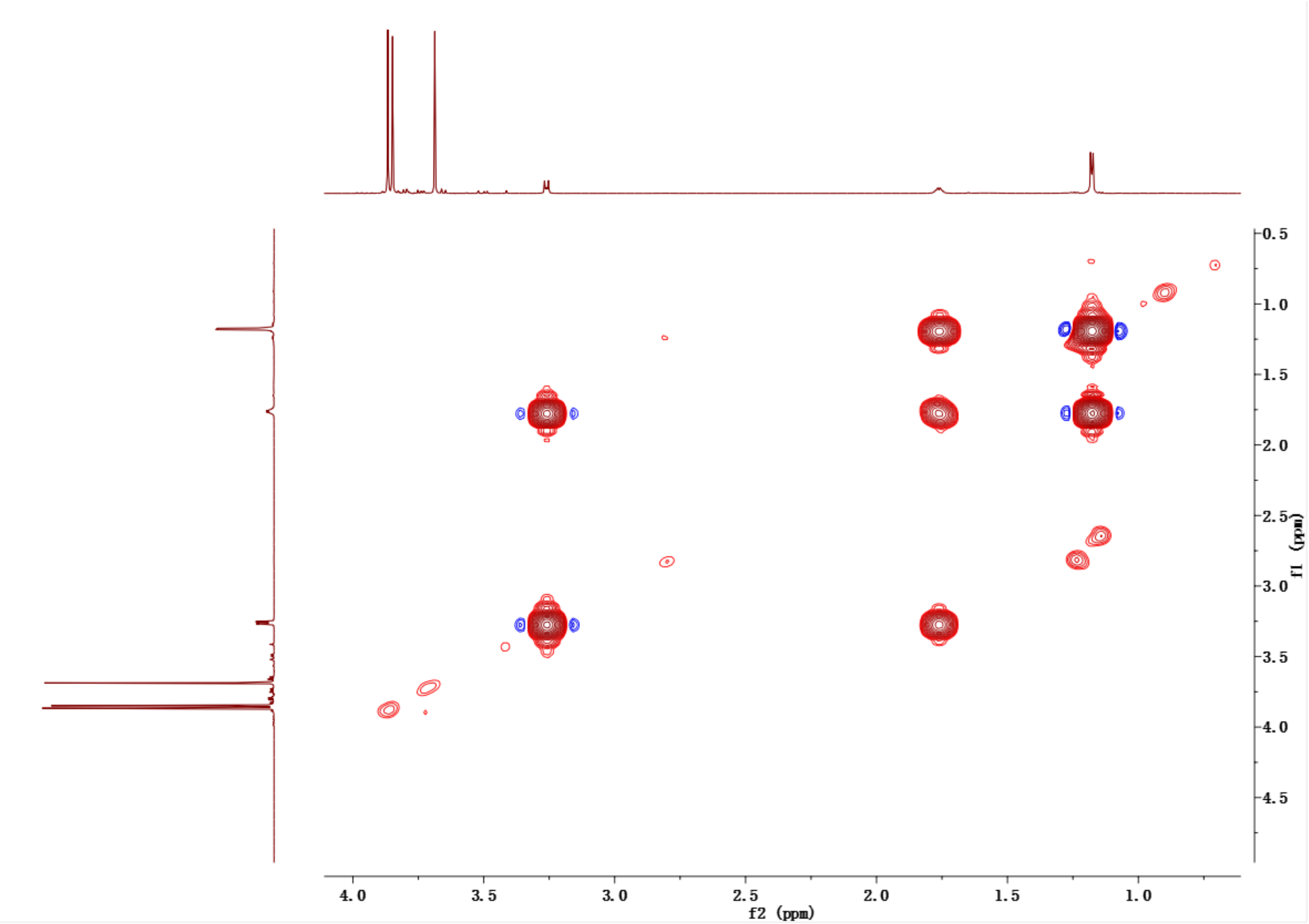

Figure S16. The ^{1}H-^{1}H COSY spectrum of compound **2** in $CDCl_3$

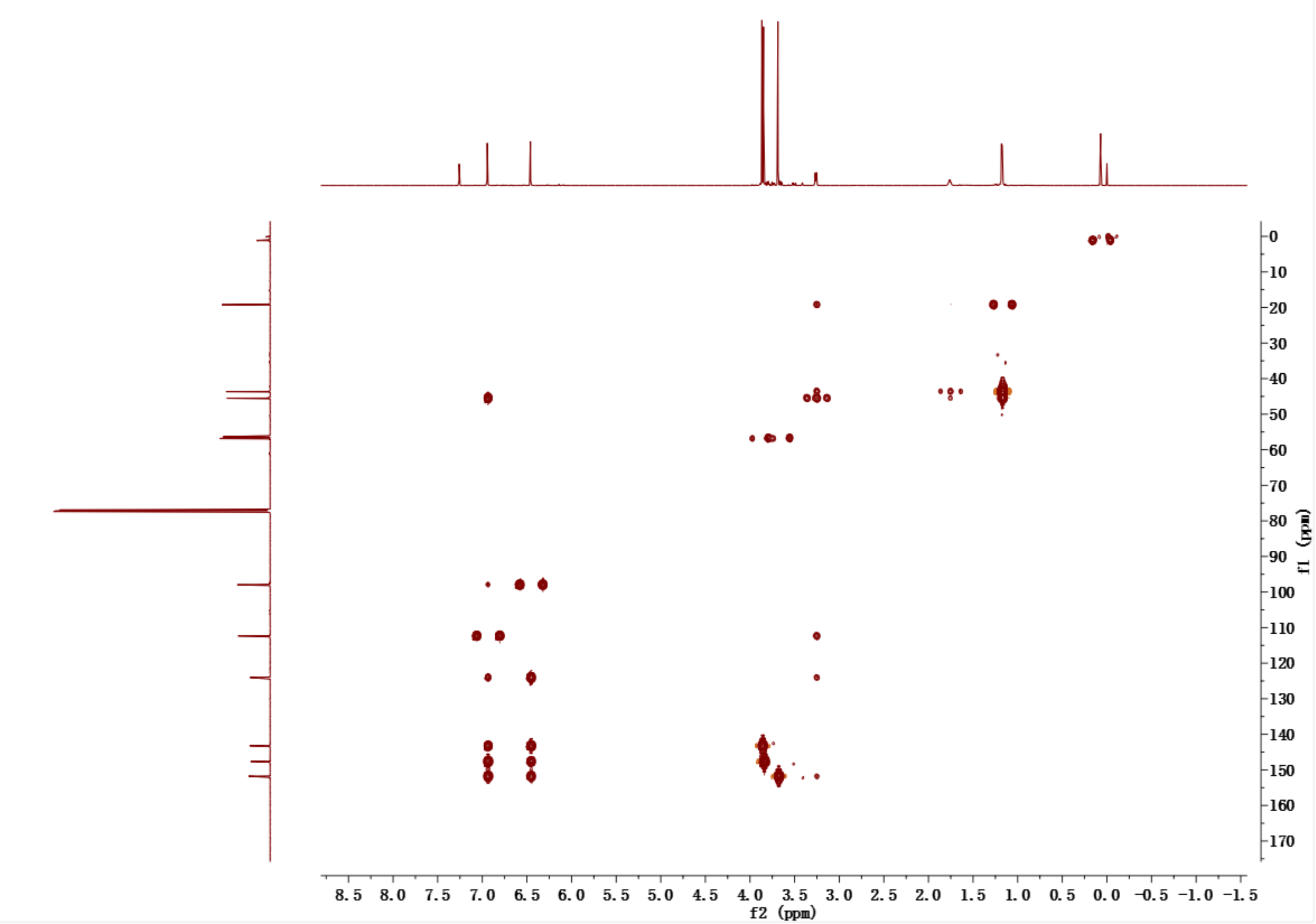

Figure S17. The HMBC spectrum of compound **2** in $CDCl_3$

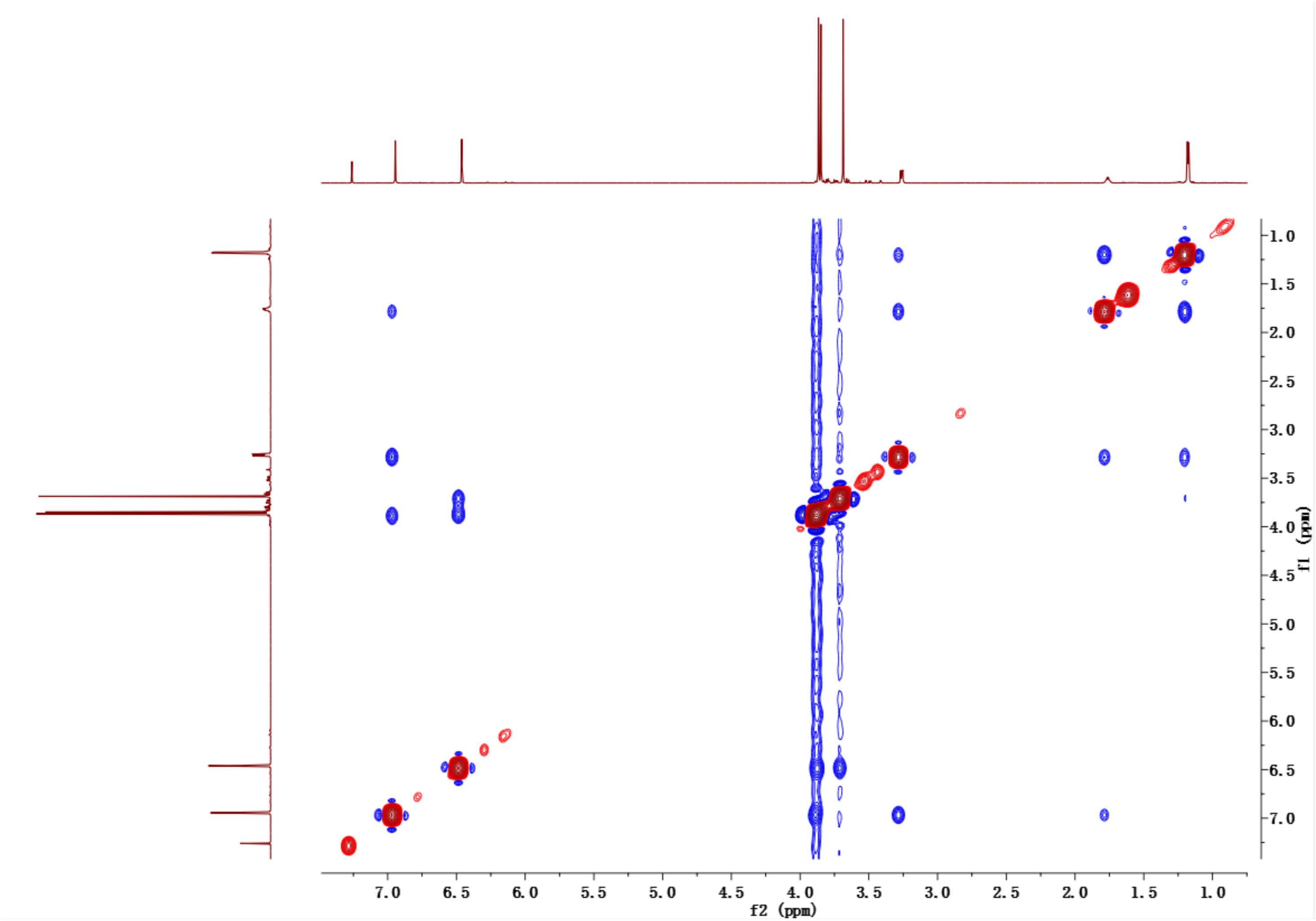

Figure S18. The NOESY spectrum of compound **2** in $CDCl_3$

Table S3. The original western blots in three repetitions for Figure 7 in the paper

	n1	n2	n3
iNOS	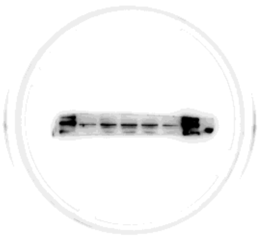	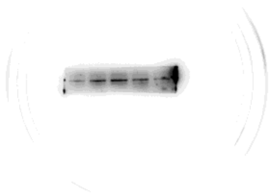	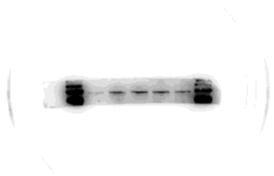
COX-2	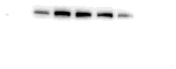		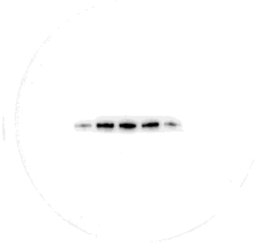
β-actin	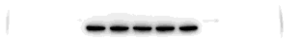	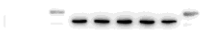	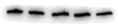
